# Reply to Soldo, A.; Lipej, L. Comment on “Balàka et al. Updated Checklist of Chondrichthyan Species in Croatia (Central Mediterranean Sea). *Biology* 2023, *12*, 952”

**DOI:** 10.3390/biology13030136

**Published:** 2024-02-21

**Authors:** Pia F. Balàka, Pero Ugarković, Julia Türtscher, Jürgen Kriwet, Simone Niedermüller, Patrik Krstinić, Patrick L. Jambura

**Affiliations:** 1 Department of Palaeontology, University of Vienna, Josef-Holaubek-Platz 2 (UZA II), 1090 Vienna, Austria; tuertscher.julia@gmail.com (J.T.); juergen.kriwet@univie.ac.at (J.K.); 2 World Wide Fund for Nature Adria (WWF Adria), Gundulićeva 63, 10 000 Zagreb, Croatiapkrstinic@wwfadria.org (P.K.); 3 Vienna Doctoral School of Ecology and Evolution (VDSEE), University of Vienna, Djerassiplatz 1, 1030 Vienna, Austria; 4 World Wide Fund for Nature Mediterranean Marine Initiative (WWF MMI), Via Po 25/c, 00161 Rome, Italy; simone.niedermueller@wwf.at

Balàka et al. [1] compiled a taxonomic list for chondrichthyan fishes in Croatian waters and discussed the spatial and temporal dynamics for each species, as well as their taxonomic validity. Soldo and Lipej [2] questioned the relevance of a national checklist for Croatia [1], as two species checklists covering the Adriatic Sea have already been published [3,4]. However, we want to emphasize that national checklists serve to support national biodiversity management, as the need to prepare a national report for Croatia for the Action Plan for the Conservation of Cartilaginous (Chondrichthyan) Fishes in the Mediterranean in 2009 demonstrates [5]. These species inventories allow the countries concerned to improve the transposition and implementation of relevant regional and global legislation into national legislation, policies, and national management measures. National checklists are generally crucial to implement a solid national species monitoring system and to track biodiversity changes over time [6].

According to Soldo and Lipej [2], the coastline of Croatia covers such a substantial portion of the Adriatic that it is not reasonable to realize a national checklist additionally to an Adriatic one. However, although Croatia has the longest coastline of all Adriatic countries, the coastline of Italy still covers 1249 km, which is one third of the entire Adriatic coast [7]. Moreover, Croatia’s coast is characterized by unique karst formations and differs significantly to other regions of the Adriatic such as the far north, where the Po Delta is situated, and to the far south, where deeper waters merge with the Ionian Sea. Still, the bathymetry, temperature, and topography of Croatia’s Exclusive Economic Zone are very diverse, and the presence of sharks, rays, and skates has been shown to be highly correlated with these environmental parameters [8]. Tracking the faunal communities of such habitat-rich countries is difficult but important to obtain an overview of the animals that live there and that are therefore subject to either exploitation or protection by the government’s national fishing and environmental protection legislation. Secondly, the size and structure of fisheries in Croatia, as well as its fish markets and fish trade, significantly differ compared to other Adriatic countries such as Italy [9], not only currently, but also in the past [10]. Therefore, having a specific, peer-reviewed checklist for Croatia, which we provided for the first time [1], is a useful and relevant tool for policy makers to make sound decisions about sharks, rays, skates, and chimaeras in Croatia based on scientific evidence [1].

The main concern of Soldo and Lipej [2] was our revision of the taxon *Carcharias taurus* Rafinesque, 1810 and our conclusion that previous records of this species are ambiguous and likely the result of misidentification with the closely resembling species *Odontaspis ferox* (Risso, 1810), which is also known to occur in Croatia [1,3,11,12].

Since the 19th century, five records of *Carcharias taurus* have been reported from Croatia [13], with only one record being supported by photographic evidence [12]. We were able to obtain an additional photo of the same specimen (Figure 7 [1]) and identified it as *O. ferox* based on characteristics related to its color pattern, dorsal fin position, and eye size, all of which have been deemed reliable taxonomic features to identify both species [14,15,16].

*Carcharias taurus* has been reported to have dark spots on the trunk and caudal fin region [14,15,16], a feature that was not present in our specimen. We acknowledge that phenotypic variation in the presence or absence of spots has been previously reported in *C. taurus* [15]. However, individual spot patterns were used to identify individuals and to study the population dynamics of *C. taurus* in Australia [17,18,19]. In these studies, hundreds of individuals were identified by their spot patterns, and not once was it mentioned that an individual lacked such a pattern. Although Castro [20] stated that the spots of *C. taurus* fade with age and disappear once the animals reach a total length of 180–200 cm, this was not reported in the studies on Australian *C. taurus* populations, which included subadult and adult animals above 180 cm [17,18,19]. The first multi-year study that examined both captive and wild *C. taurus* on the persistence of spot patterns observed that “spot numbers, positions and sizes did not change”, even over a period of 14 years [18]. The authors of the study concluded that since the unique spot patterns do not change over time, they are suitable for individual shark recognition, also in older individuals [17,18,19]. This, of course, cannot serve as proof that there are no *C. taurus* individuals lacking these spots at all or losing them with age, but given the apparent rarity of such a phenomenon, it seems unlikely that the only photographic evidence of *C. taurus* in Croatia shows such a rare case. Nonetheless, we agree that coloration alone should not be used for species identification, but in combination with the characters discussed in our original article [1], we are confident that coloration can provide supportive evidence for correct species identification.

An additional taxonomic feature that supports our identification is the position of the first dorsal fin in relation to the pectoral and pelvic fins. In *C. taurus*, the first dorsal fin is situated closer to the pelvic fins than to the pectoral fins, whereas in *O. ferox*, the first dorsal fin is closer to the pectoral fin bases than to the pelvic fin bases [14,15,16]. Soldo and Lipej [2] argued that the claim to be able to accurately measure the distances to the pelvic and pectoral fin was “arbitrary”, as the dorsal fin was not clearly visible on the photographed specimen. Even though we never claimed to provide accurate measurements, the origin of all fins was clearly visible and marked on the provided image, demonstrating that the origin of the first dorsal fin was immediately after the pectoral fins and thus not consistent with the position of this structure reported in *C. taurus*, but with that of *O. ferox*. Curiously, despite stating that the dorsal fin was not clearly visible in the provided figure (nor in the figure in the original reference [12]), Soldo and Lipej [2] criticized us for not using the relative size of the two dorsal fins in relation to each other (in *C. taurus*, both are of similar size, whereas in *O. ferox*, the first dorsal fin is noticeably larger [14,15,16]), which are apparently of the same size in the photographed specimen according to them. However, the dorsal fins in the picture are turned sideways, making it impossible to evaluate the height of the dorsal fins in any of the published images, and it therefore remains unclear how the authors of the commentary came to this conclusion.

Another feature we mentioned was the size of the eyes, which are comparatively larger in *O. ferox* (1.6 to 2.8% of the total length) than in *C. taurus* (0.9 to 1.4% of the total length) [15]. Digital measurements of the axial diameter of the eyes of the specimen discussed here indicate that the eyes are approximately 1.8% of the total length and would thus fall within the range of *O. ferox*. However, these measures can only be regarded as approximate values, as no exact measurements could be taken since the specimen in the picture is not positioned exactly parallel to the camera [1]. Nonetheless, the eyes provide an additional distinguishing feature, namely the color of the iris, which has been reported to be light green in *C. taurus* and black in *O. ferox* [15]. The illustrated specimen clearly shows a large, black iris, further supporting its identification as *O. ferox*.

Finally, one more character supports our identification of the specimen as *O. ferox*: the total length. Lipej et al. [12] stated that the total length of the specimen was about 380 cm. However, the authors write in the same book that the maximum length of *C. taurus* is 318 cm, while *O. ferox* can reach up to 410 cm [12].

Despite all the evidence pointing towards *O. ferox*, Soldo and Lipej [2] insist on identifying this species as *C. taurus*. According to them, body measurements underlie too much variation and the only reliable taxonomic character for delimiting these species are the teeth and jaws. While dental characters provide valuable taxonomic information, we caution against overstating their significance. A wide range of intraspecific variation has been reported in a number of species, including (but not limited to) variable tooth numbers, tooth morphology, tooth polarity, presence and absence of teeth in certain positions (e.g., symphyseal teeth, intermediate teeth, etc.), and the number of cusplets [21,22,23]. According to Lipej et al. [12], the number of lateral cusplets is the main dental feature to distinguish between *C. taurus* (one pair of dental cusplets) and *O. ferox* (two pairs of dental cusplets) [12]. However, the variability in this trait is possibly best demonstrated by a recent observation of a specimen of *Odontaspis noronhai* (Maul, 1955) from the northwestern Pacific [24]; while the number of lateral cusplets is also regarded an useful distinguishing feature between *O. ferox* (two pairs) and *O. noronhai* (one pair), 44 out of 58 teeth in this specimen exhibited two pairs of cusplets, while the remaining teeth exhibited one pair of cusplets, demonstrating that both conditions can occur within the same individual. The identity of this species was confirmed by molecular data [24]. These examples are not meant to reflect our skepticism about the taxonomic significance of teeth, but merely to illustrate that these morphological structures exhibit the same variability as other morphological characters. However, even under the assumption that the number of cusplets is constant and not affected by variability, the images of the jaw of this specimen (Figure 11 in Lipej et al. [12]) were of poor quality, and did not allow us to identify any relevant dental characters. Our request to the authors to provide us with the original image was unfortunately declined.

The presence of *Odontaspis ferox* in the Adriatic Sea is already well established, as there is undoubted evidence for the occurrence of this species in these waters [1,3,11,12]. All morphological features that are visible in the photograph support the identification of this specimen as *O. ferox*. As this was the only photographic evidence for *C. taurus* in the Adriatic Sea, we remain cautious regarding the occurrence of *C. taurus* in this area. We agree with Soldo and Lipej [2] that in the absence of verifiable evidence, the identification of species and subsequent publication of such “arbitrary observations” should be avoided, and we therefore consider the proposed records of *C. taurus* along the Croatian coast to be doubtful until robust evidence, preferably based on both morphological and molecular data, is available to suggest otherwise.

In their commentary, Soldo and Lipej [2] stress the difficulties regarding *Scyliorhinus duhamelii* (Garman, 1913), which we already described in detail in our paper [1]. *Scyliorhinus duhamelii* was regarded as a junior synonym of *Scyliorhinus canicula* (Linnaeus, 1758) but was recently resurrected and proposed to be a valid species [25,26]. Despite the ongoing discussion, the fact that it was not included in previous checklists for the Adriatic Sea is no reason to exclude it from any updated checklist. In our paper, we included this species with caution, indicating that the validity of this species is still discussed and needs further confirmation. We eventually included it in our list in order to provide an outlook for the scientific community and to initiate a discussion about its validity and occurrence in the Adriatic Sea.

The population genetic analyses of *Scyliorhinus canicula* performed by Gubili et al. [27] strongly support the hypothesis of a Mediterranean catshark species separate to an Atlantic one that split from a common *S. canicula* population; the clustering analysis of microsatellite data of *S. canicula* individuals divided the samples into two distinct clusters—one from the Atlantic and one for Sardinia, Crete, and the Adriatic—whilst the samples from Mallorca showed a transitional state between these two clusters [27]. Also, the mitochondrial DNA data analysis of the study found a number of haplotypes that were unique to the eastern Mediterranean Sea, with a significant genetic differentiation amongst Mediterranean populations, whilst Atlantic populations were genetically homogenous [27]. The study found a significant relationship between genetic and geographical distance across all sample sites and found a significant pattern of isolation by distance (IBD) in the catshark species *S. canicula* [27].

Cryptic shark species are known to have relatively low levels of genetic differentiation [28,29,30,31,32,33,34,35,36], which might be the reason why previous population genetic studies of *C. canicula* were cautious to suggest a species distinct to *S. canicula*, although all of them found strong genetic differentiation [27,37,38]. Future studies might require a whole-genome-wide sequencing approach to cover as much genetic depth as possible [34].

Soares and Carvalho [26] described the distinct diagnostic morphological characters of *Scyliorhinus duhamelii* that now make it possible to distinguish the species from the closely related *S. canicula* in the field [25]. Future studies will require a combination of morphological analyses and genetics to determine whether the genetic regional subclusters observed by Gubili et al. [27] correspond to individuals of *S. canicula* or rather *S. duhamelii*. As morphological, ontogenetic, and genetic differences between Atlantic and Mediterranean populations of *S. canicula* have been observed for a long time now [27,37,38,39,40,41], future studies will be necessary to shed light on possible cryptic catshark diversity in the Mediterranean Sea, as we stressed in our paper [1].

Soldo and Lipej [2] questioned the inclusion of *Squatina aculeata* Cuvier, 1829 in the taxonomic list, as it has not been reported by any other Adriatic species list yet [3,4]. Again, we have to highlight that the exclusion of a species from previous checklists is not in itself a reason to exclude it from future checklists, given that enough scientific evidence for its (past) occurrence in an area is provided. We critically discussed a specimen housed in the collection of the Natural History Museum Vienna (NHMW), for which we provided detailed pictures and morphological descriptions, which gave us sufficient reason to add it to the list. Also, the presence of this species in Croatia is further supported by an additional record [42], even though that specimen has been unfortunately lost. This makes the record from the NHMW even more important, as it serves as a voucher for the occurrence of this species in the Adriatic Sea [1].

Soldo and Lipej [2] expressed their confusion about the status of *Sphyrna tudes* (Valenciennes, 1822), as it was listed in Table 1 but discussed to be unconfirmed. It is true that we included *S. tudes* in the species list table, but we marked it with two asterisks, indicating that this report is unconfirmed [1]. We hope that this clears up the confusion expressed by Soldo and Lipej [2].

Finally, in their commentary, Soldo and Lipej [2] pointed out two references [43,44] that were related to the most recent observations of three chondrichthyan species (*Chimaera monstrosa* (Linnaeus, 1758), *Raja asterias* Delaroche, 1809, and *Raja radula* Delaroche, 1809) but missing in our bibliography. It is the aim of any study to provide a complete list of references, a goal we pursued by compiling historical, citizen science, and literature data, which to date represents the most comprehensive list of chondrichthyan occurrences in Croatia [1]. We are grateful for the additional references provided by Soldo and Lipej [2], which further enhance the richness of our compilation.
